# Medicine Non-Adherence: A New Viewpoint on Adherence Arising from Research Focused on Sub-Saharan Africa

**DOI:** 10.3390/healthcare12080860

**Published:** 2024-04-19

**Authors:** Peter Michael Ward

**Affiliations:** Service Systems Research Group, WMG, University of Warwick, Warwick CV4 7AL, UK; p.ward@warwick.ac.uk

**Keywords:** medicine, adherence, sub-Saharan Africa

## Abstract

Adherence is vital for medicine to have an effect, yet adherence is considered to be low, with approximately half of the patients not fully adherent. However, research into adherence tends to focus on quantitative analysis of performance, which fails to perceive how people are adherent in their many different environments. As a contribution to gaining a deeper understanding, interviews were held with thirty individuals in the UK, Egypt, Kazakhstan, and six countries in sub-Saharan Africa to understand their perceptions on adherence to a range of drugs, and these were compared with an existing well-regarded list. New or undocumented reasons for non-adherence were discovered. Reasons for non-adherence were consistent across both developing and developed worlds. A new viewpoint on adherence is suggested, which considers adherence to be a single act and therefore as an individual opportunity to be adherent, permitting greater focus on the enablers and inhibitors of adherence at any given point in time.

## 1. Introduction

In his seminal 2003 report for the World Health Organisation (WHO), Sabaté [1] (p. xiii) said, “[Increasing adherence] may have a far greater impact on the health of the population than any improvement in specific medical treatments”. Adherence to instructions for medicine consumption is a fundamental requirement for health. Indeed, McColl-Kennedy et al. [2] refer to it as “Comply[ing] with basics”, yet non-adherence is a significant worldwide issue. For example, it has been estimated that 125,000 people die each year just in the USA as a result of non-adherence [3]; figures for other parts of the world are not known. In the developed world, half of the patients are not fully adherent to their prescription instructions [1,4,5], and it is thought that the proportion of non-adherence is higher in the developing world [1].

A significant amount of practical research has been performed on the issue of adherence [1,5]. Peterson et al. [6] found 95 studies on adherence. More recently, a narrative review [7] identified a total of 38 systematic literature reviews of adherence papers. A recent search of the MEDLINE database for the term “medicine adherence” revealed that almost 19,000 such papers have been published.

**Figure 1:**
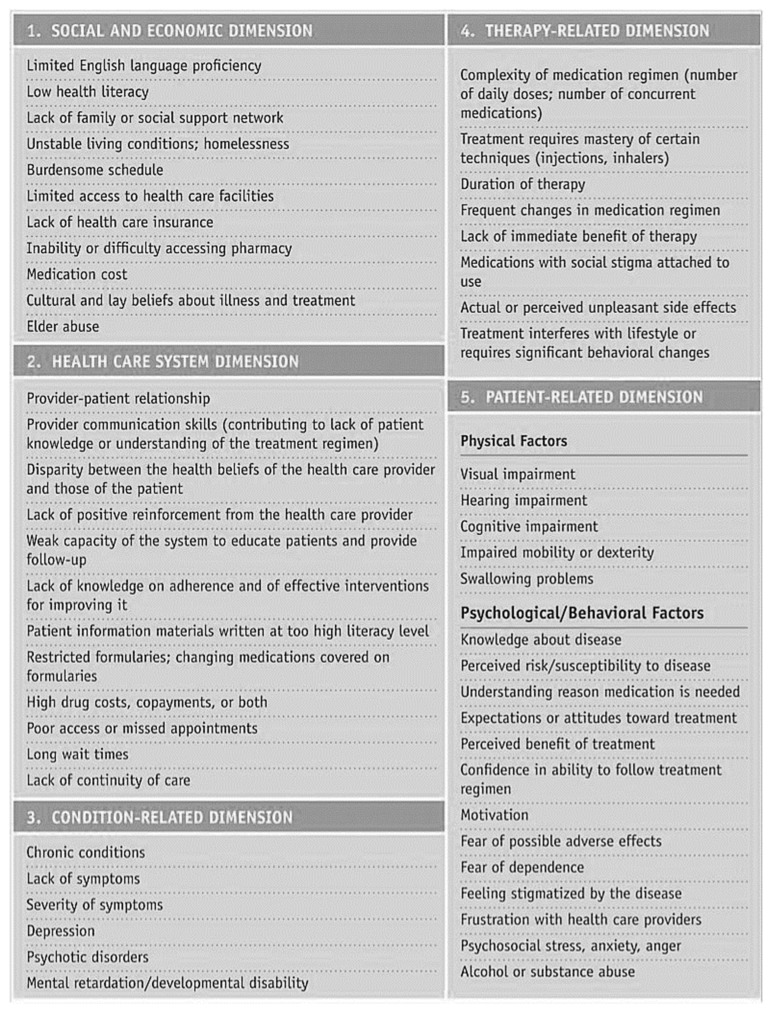
The 55 causes reported to affect adherence [8]. Table republished with permissions from the American Society on Aging and American Society of Consultant Pharmacists Foundation.

Sabaté’s World Health Organisation report is a milestone in the field. Building on his work, another empirical report, “Adult Meducation: Improving Medication Adherence in Older Adults”, produced jointly by the American Society on Aging and the American Society of Consultant Pharmacists Foundation [8], categorised 55 causes of non-adherence using the five “dimensions” of Sabaté’s report: health system/HCT, social/economic, therapy-related, and patient-related and condition-related factors; see Figure 1.

There are limitations to the practical research performed so far. Firstly, most research has had a primarily Western focus and may not be completely applicable in the developing world. Secondly, there has been a concentration on age-related issues in the USA and HIV/AIDS-related issues in sub-Saharan Africa. It is, therefore, possible that further important information on the causes of non-adherence, including details that may be specific to particular medicines or be geographically localised, still remains to be captured.

This study investigates people’s experiences of adherence in their lived lives, with the aim of exploring reasons for non-adherence and identifying new causes not documented so far. A series of semi-structured interviews was arranged with people who were willing to talk about their past experiences of taking medicines. They were located in various environments ranging from a comfortable urban environment in a developed country through to an impoverished rural environment in a developing country.

## 2. Materials and Methods

Interviewees were selected using purposive and snowball sampling [9]. Initial interviews were performed with six contacts in the UK to explore the situation in the developed world. Following that, twenty-four interviews were arranged with contacts in Kenya, Tanzania, Zambia, Zimbabwe, Uganda, Nigeria, and Kazakhstan. These were intended to explore the developing world, primarily sub-Saharan Africa. A total of thirty interviews were conducted over a period of just over five months from the end of December 2014 to early June 2015.

Semi-structured interviews were performed, either face-to-face or by telephone. Interviews generally lasted for 25–30 min. Table A1 and Table A2 in Appendix A summarise the interviewees. All interviews were performed by the author in English. All those asked were willing to be interviewed and gave their approval via reading a Participant Information Leaflet and agreeing to the terms of a Consent Form. The questions asked are listed in Table 1.

Each interview was recorded and transcribed. A combination of Nvivo and manual means was used to code the transcripts. The general approach of Systematic Combining [10,11] was used to revise the initial framework based on empirical findings. Codes were analysed and a taxonomy of non-adherence was created. Further analysis was performed to compare the reasons for non-adherence discovered in interviews with the list of 55 reasons from the “Adult Meducation” report [8].

## 3. Results

### 3.1. Coding Categories

Table A3 and Table A4 in Appendix B show phrases extracted from interview transcripts and how they were coded, looking separately at the developing and developed worlds.

Some examples of coding are as follows:

- Interviewee EG01 said, “…pharmacies in every street… just down the road from our flat”, and this was counted as “Distance, Positive, Close”, while interviewee UG01 said, “It’s 30 km to and from, to the pharmacy. USD 10 [GBP 6.57] transport” which was considered to be “Distance, Negative, Far”
- Interviewee NG01 said, “Sometimes I’ll take it according to the prescription but sometimes I stop when I feel better”, which was coded as “Stop, Negative, Better”, while interviewee KN03 said “They act like emergency for my family” which was coded “Stop, Negative, Keep”
- Interviewee KN08 said, “This tablets are in large sizes and so swallowing becomes a problem”, coded as “Size, Negative, Big”.

In this way, all relevant interview statements were captured and coded. Table 2 shows the coding derived from the interviews. As can be seen, not all categories have positive as well as negative attributes, but the focus of the interviews was on non-adherence and so this is to be expected.

As part of this work, surprises were found regarding the overall approach to adherence on the part of some interviewees. For example, some stopped taking medicine when they felt better even if it was an antibiotic; many struggled with tablets being too big to swallow or possessing a bitter taste; one commented on how the pharmaceutical industry was making profits from medicines; several were afraid of rumoured side effects. There was a wide spread of reasons for why adherence was not achieved.

### 3.2. Taxonomy

It proved possible to consolidate these reasons. Further analysis was performed to create a taxonomy of non-adherence categories, identifying five entities relating to non-adherence. Table 3 summarises this.

In line with normal usage, in this analysis, “agency” refers to the capacity of individuals to have the power to fulfill their potential, “affordance” is a property of an object that determines how it might be used, and “context” is the situation within which something exists or happens.

This taxonomy shows that motivation is just one cause of non-adherence, despite being the one that receives strong focus. There are more reasons for non-adherence relating to the medicine than there are to the patient, while the consumption context is critical to adherence. Summarising this, from Table 3, it can be seen that there are three factors at play in adherence: patient, medicine, and context.

### 3.3. Reasons for Non-Adherence

As well as identifying these three factors, the reasons given for non-adherence were assessed against the list of 55 in the “Adult Meducation” report [8]. Ten causes in the report were not mentioned in the research. These were of the type where the interviewee would have to expose themselves to what may be considered an unacceptable degree or which needed to be inferred by the interviewer in a face-to-face situation. Examples are “Mental retardation” or “Alcohol or substance abuse”.

Table 4 shows the 19 reasons for non-adherence discovered in interviews which were not mentioned in the report [8]. While some of these might be obvious and anecdotally known, they have not been documented in formal research to date.

Similar causes of non-adherence were seen in both the developed and developing worlds. For example, a lack of food and water for taking tablets was referenced in both, yet this was not mentioned in the list of 55 causes. This suggests that interviews are of significant importance both to understand non-adherence reasons in detail and also to expand the list of known reasons.

## 4. Discussion

The qualitative research results have provided a rich view of adherence as part of people’s lived lives in a range of environments from extreme poverty to relative comfort, across both developed and developing worlds. The results have extended our understanding of the phenomenon of non-adherence and provided insights into the range of causes beyond prior knowledge.

### 4.1. Broadening the Scope of Adherence Research

The categories derived from the interviews provide a valuable picture of the broad spectrum of factors which make up adherence in context. The taxonomy of entities leads to the conclusion that to understand adherence, we must consider the three aspects of patient, medicine, and context together. It has not previously been normal to bring all three of these into research at the same time.

For example, it is clear that motivation is an important part of adherence, yet it is just one factor among very many. The focus on increasing motivation in a lot of adherence interventions is potentially missing the wider perspective. Even simply considering patient agency and beliefs broadens the scope of intervention. Based on this research, considering agency as relevant to adherence would bring into view the topics such as the length of a course, the imposition of the regimen on the patient’s routine, and the causes of stopping. Would it be possible to shorten the course or to reduce the number of doses per day? This would be an intervention on the product side which reduces the need for patient agency, thereby facilitating adherence.

Taking context and medicine into account could make an even more significant impact. Consumption context is a potential major area of investigation. This research identified seven categories of causes of non-adherence under the heading of context (Table 3): people, utensils, reminder, water, food, storage, and norms. Norms is a large area, raising questions of culture that then includes the effects of stigma on medicine consumption. But the issue of utensils, for example, could simply be addressed by providing a suitable spoon with the medicine.

The medicine itself is perhaps the area that could generate the largest potential improvement in adherence. Product affordance was a factor in thirteen categories of non-adherence including taste, size, and smell (Table 3). These could be addressed relatively simply by manufacturers if they were to take the issues seriously. Others might be more challenging but taking them seriously as causes of non-adherence could pay dividends.

### 4.2. Non-Adherence Reasons

The “Adult Meducation” report [8] documented 55 causes of non-adherence. This research uncovered 19 more. Many causes were seen in both developing and developed worlds, indicating that although root causes of non-adherence might be different in some cases, their manifestations are the same, for example, a lack of water, a lack of food, keeping medicine for future use, or misunderstanding the instructions.

Some causes of non-adherence would not routinely be considered in the developed world, for example, a dislike of supporting the pharmaceutical industry’s profits, or concern that the medicine is foreign. However, it makes sense to consider shared causes because interventions might be globally valuable or make a particular contribution to poorer areas, such as keeping medicine for future use or for family needs. This implies that price and availability are relevant, but also, in consideration of “feeling better”, a lack of understanding that some medicines must be consumed until the prescription is complete. As well as patient education, this implies the importance of providing clear instructions in a language that the patient understands and that is consistent in both written and verbal forms.

It may be seen that some of the factors of non-adherence are interrelated and can be traded off against each other. For example, if the affordance of the medicine is perceived by the patient as being inadequate in itself to permit adherence to take place, they may be able to call on other resources from context and agency to overcome such inadequacy. If the medicine is bitter, then the patient may be able to use their agency to bring sugar into context to sweeten it. If it requires food to be eaten at the time of consumption and there is none available, then support may be obtained from an alternative source. These simple examples demonstrate the potentially complex interactions between adherence factors.

Some adherence factors are effectively “mirror images” of each other. For example, a patient’s context may not be contributing sufficient resources to permit adherence, but if the medicine’s affordance were to be enhanced then consumption might still be able to occur. Perhaps a patient’s context cannot provide food or water, but if these could be incorporated into the medicine in some way then the patient may still be able to be adherent. Similarly, the patient’s agency may be limited—perhaps not being able to open the bottle or swallow large pills—but enhancements to the medicine might address such limitations.

### 4.3. Unit of Analysis of Adherence

One important facet of this research is the focus on adherence as an individual act rather than an average of all adherence events for a single patient or even a cohort of patients. This approach has highlighted reasons for non-adherence rather than just measuring it.

A lot of research on intervention highlights the limited impact that interventions achieve. For example, when van Dulmen et al. [7] reviewed 38 systematic reviews, they discovered that only 45% of interventions resulted in improved adherence, and only 33% in improved outcomes. Many papers discuss the need for, or evaluation of, multiple forms of intervention to improve adherence rates. This is discussed in two reviews [6,12]. Kardas et al. [12] suggested in their review that “multifaceted interventions may be the most effective answer”, but at the same time, they found that many of the reviewed papers reported mixed or limited success (for example [13,14,15]). Without an understanding of adherence enablers and inhibitors in patients’ lived lives such as has been discerned in this research, it is not surprising that interventions have limited impact.

### 4.4. Intention and Reality

When adherence research incorporates a theoretical perspective, it tends to use expectancy-value models, usually the Theory of Planned Behaviour [16,17], for example [18,19]. The limitation of such theories is that they reach only as far as the intention to act. They hold an implicit assumption that intention leads directly to behaviour, overlooking the possibility that it is not always true. This research has demonstrated that motivation—the intention to act—is just one element of adherence and that there are many factors that can prevent it, including those relating to the medicine and operating within the consumption context. A new theory of medicine adherence is required which recognises this in order to make progress towards higher adherence levels.

## 5. Conclusions

### 5.1. The Triad of Adherence

It is normal in adherence research to consider dyads. There is the dyad of prescriber and patient, for example. But this research has brought out the importance of considering the whole picture of the triad of the patient and medicine in a consumption context. Looking at all three aspects allows the full picture of adherence to be seen. Understanding the three aspects and how they interact with each other as a system provides insights into reasons for non-adherence that cannot otherwise be discerned. This approach has uncovered new reasons for non-adherence.

### 5.2. Reasons for Non-Adherence

Nineteen new reasons for non-adherence were documented as a result of this qualitative research. At a time when much of the adherence research is quantitative, assessing adherence by percentage compliance with instructions, it is important to understand that people have multiple reasons for their non-adherence which cannot be captured quantitatively. If we are to help people to become more adherent, we need to understand their circumstances. Putting all non-adherence down to a lack of motivation misses the point that this is just one of many facets. A deeper understanding of people’s lived lives can identify interventions which might make a difference to compliance.

Reasons for non-adherence were remarkably consistent across the developing and developed worlds. Though caused differently, the outcomes were the same. For example, a lack of water at the time of consumption was identified in both sub-Saharan Africa and the UK as a cause of non-adherence.

### 5.3. Adherence as a Point-in-Time Opportunity

Considering all this, it can be seen that adherence is not a percentage figure but is achieved or otherwise each time consumption is due. It is either 100% or 0%. Understanding the point-in-time reasons for non-adherence will permit actions to be taken which increase the number of times when adherence is achieved, thus enhancing the effectiveness of interventions.

For example, sometimes water is not available and adherence cannot be achieved. Reformulating the medicine so that water is not a corequisite will address this cause of non-adherence. It may only be effective one time in ten but at that time it makes a 100% difference in adherence. Viewing adherence as a percentage of all consumption opportunities may overlook this point.

### 5.4. Learning for the Pharmaceutical Industry

The points mentioned above suggest that medicine formulations might be more intelligently designed, and that this might benefit people worldwide. A lack of water to consume a tablet in Kenya might be due to there being no water in the well, but a lack of water in the UK could be that the patient is a passenger in a car. Whatever the cause, non-adherence is the result. What steps can be taken to remove the requirement of water from the consumption context? Can the medicine be provided in another formulation, perhaps? Can water be provided with the medicine? The first question relates to the manufacturer, while the second could be answered at the pharmacy. They could be long-term and short-term answers or could depend on the medicine.

Considering some of the other reasons for non-adherence, we might apply the same line of thinking to the subject:

1. Lack of food: Can food be provided with the medicine? Can the active ingredients be incorporated into some form of food?
2. Bad taste: Can the medicine be sweetened in some way? Can the taste be masked?
3. Large size: Can the tablet size be reduced? Can the formulation be changed?
4. Bad smell: Can the formulation be changed? Can the smell be masked?
5. Lack of dosing spoon: Can a spoon be provided in the medicine packaging or by the pharmacist? Can the formulation be changed?

Considering the other categories identified, it seems reasonable to explore what the pharmaceutical industry can do to address medicine affordances in all the identified areas of content, branding, effects, taste, formulation, size, smell, instructions, regimen, distance, access, cost, and diagnosis. It may contribute to some of the contextual categories of people, utensils, reminder, water, food, storage, and norms. In particular, medicines which more completely address contextual challenges could be more successful in raising adherence than those which at present might be perceived as “one size fits all” or even “lowest common denominator”. Some factors will prove to be out of the manufacturers’ scope and perhaps more related to healthcare providers and pharmacies, but others might be easily tackled once they become the subject of some analysis.

Patient centricity is a goal for many in the industry, and taking this approach could enhance that focus. Using the insights gained from in-depth qualitative research could deliver new ways of supporting patients to be adherent, moving towards the goals of increased adherence and higher quality of life.

### 5.5. Research Limitations

The research was performed remotely. A more ethnographic approach might have both confirmed the remaining 10 causes of non-adherence present in the “Adult Meducation” report [8] that were not found in the research, and potentially uncovered additional causes through observation and interviews with family members, medical staff, etc.

Interviews in some countries were limited to just one. Further information may have been obtained with a greater number of interviewees per country.

This research considered only one developing country, the UK. Although this was not a focus of the research, which primarily addressed reasons for non-adherence in sub-Saharan Africa as a representative area of the developing world, investigation in other developed countries might have provided a richer picture of non-adherence reasons.

### 5.6. Opportunities for Further Research

It would potentially be useful to perform further qualitative research face-to-face with interviewees in their contexts. This should reveal a greater depth of insight and add further understanding of non-adherence in sub-Saharan Africa.

The same approach could be taken to explore adherence to products other than medicine. For example, a fitness regime or a smoking cessation course also requires the participants to be adherent. Considering adherence as a point-in-time opportunity would allow researchers to study the triad of the patient and the “product” in context to understand non-adherence in more detail.

Theoretical work on the development of a theory of adherence could pay dividends in increasing adherence. It would start from the position of recognising the complex dynamics operating between the elements of the triad of adherence and go beyond the focus on motivation to consider the holistic picture. Viewing adherence as a (complex) process where patient agency and medicine affordances come together into a consumption context would permit a deeper understanding of the interactions of the non-adherence categories in enabling or preventing adherence [20].

## Figures and Tables

**Table 1:** Semi-structured interview questions.

Number	Question
1	What medicine do you wish to share your experiences of?
2	Is this your first time with this medicine or is it a repeat prescription?
3	How far was it to a pharmacy?
4	How much did it cost you to buy the medicine?
5	Did you obtain the medicine?
6	If you obtained the medicine, how did you feel about it at the time?
7	Did you actually plan to consume it in line with the prescription?
8	Did you know how to take this medicine? How do you know?
9	Please describe your physical surroundings on various occasions when the prescription said you should consume. Who and what was there and not there?
10	What were you thinking and feeling?
11	How were your physical and mental health?
12	Did you actually consume at that time?
13	What helped you to consume or prevented you from consuming?
14	Is there anything about the medicine that makes it hard for you to take it? What would make it easier for you?
15	If you had the choice, how would you like to take this medicine?
16	Anything else you want to say about what makes it easy or difficult to take medicines for you personally?

**Table 2:** Coding of interviews grouped by category.

Category	Positive Attributes	Negative Attributes
Distance	Close	Far
Access	Easy	Hard
Cost	Low	High Herbal, low
Diagnosis		Foreign language, verbal
Instructions	Clear, verbal Clear, written	Foreign language, verbal Foreign language, written Unclear, verbal Unclear, written Misunderstood
Utensils		Missing
People	Present	Absent
Content		Unknown
Norms		Others, stigma
Branding	Known	
Beliefs	Others Confidence	Others, too dependent Lack of faith Foreign origin Profit, pharma Profit, herbal Value Pointless
Motivation	Last resort Stay well Get well	Tired
Stop		Keep Replaced by other Discarded Better Busy Run out
Effects	Others Side, none	Others Side, general Side, specific Bad
Taste	Sweet	Bad Bitter
Formulation	Tablet Liquid Injection	Injection
Regimen	Acceptable	Unexpected Unacceptable Complex Forgot
Reminder	General Alarm	
Water	Present	Absent
Food	Present	Absent
Size	Small	Big
Smell		Bad
Course	Acceptable	Long
Routine	Present	Absent
Storage		Unsafe

**Table 3:** Taxonomy of categories of non-adherence.

Taxonomic Entity	Categories
Patient motivation	Motivation
Patient agency	Course, routine, and stop
Patient beliefs	Beliefs
Consumption context	People, utensils, reminder, water, food, storage, and norms
Product affordance	Content, branding, effects, taste, formulation, size, smell, instructions, regimen, distance, access, cost, and diagnosis

**Table 4:** Reasons for non-adherence beyond those documented in “Adult Meducation” [8].

Reason	Seen in Interview
Concern with medicine content	EG01
Verbal instructions in a foreign language	EG01
Written instructions in a foreign language	EG01
Pharmaceutical industry profits	EG01
Herbal medicine industry profits	EG01
Feeling better	KN03 UK05 TZ01
Lack of food	KN03 KN04 TZ01
Lack of water	KN08 UK01
Concern that medicine is of foreign origin	NG01
Lack of faith leading to need for medicine	TZ02
One medicine being replaced by another	KN03
Medicine kept for future occasions	KN03 NG01 TZ01 UK05
Medicine kept for family need	KN03 NG01 TZ01
Instructions misunderstood	UK01 KN05
Difference between written and verbal instructions	KZ01
Lack of routine	UK01
Lack of safe storage	TZ04
Forgetfulness	KZ01 TZ03
Run out of medicine	UK04

## Data Availability

Data are contained within the article.

## Appendix A. List and Summary of Interviewees

Local costs were converted to UK pounds in December 2015.

**Table A1:** Interviewee details.

Ref.	Sex	Age	Country	Medicine (Name as Given by Interviewee)	Cost	Location	Distance
EG01	F	20–40	Egypt	Cough medicine		City	Close
KN01	M	20–40	Kenya	Antibiotics	£0.03	Village	
KN03	M	40–60	Kenya	Amoxycilin		Village	1 km
KN04	M	20–40	Kenya	Malaria tablets	£3.23	Village	5 km/£2.59
KN05	M	60+	Kenya	Coartem	£0.13	City	Close
KN06	F	20–40	Kenya	Malaria tablets		Town	Close
KN07	M	20–40	Kenya	Pain killer, curatives	£0.66	Village	Close
KN08	M	40–60	Kenya	Malaria (AL)	£0.97	Village	2 km
KN09	M	20–40	Kenya	Panadol	£0.84	Village	2 km
KN10	M	40–60	Kenya	Chrotin B	£1.29	Village	6 km
KN11	F	20–40	Kenya	Quinine	£2.91	Village	2 km
KN12	F	20–40	Kenya	Flugone	£1.29	Village	3 km/£1.94
KN13	M	40–60	Kenya	Cold Cups	£0.32	Village	1 km
KN15	M	20–40	Kenya	Ibuprofen	£1.62	Village	2 km
KS01	F	20–40	Kazakhstan	Repronact	£2.09	Village	3.5 km
NG01	M	40–60	Nigeria	Artesunate	£1.49	Town	Close
TZ01	M	40–60	Tanzania	Coartem		Village	4 h
TZ02	M	60+	Tanzania	Paladrin	£1.53	Town	Close
TZ03	M	60+	Tanzania	for Stomach Abscess	£0.31	Town	Close
TZ04	F	40–60	Tanzania	Malafin, Panadol, Maleratab	£1.53	Town	10–15 min
UG01	M	40–60	Uganda	Quinine	£3.95	Village	30 km/£6.59
UK01	F	<20	UK	Roacutane, Erythromycin	Free	Village	5 km
UK02	M	40–60	UK	multiple	Free	Town	1 km
UK03	F	>60	UK	Metformin	Free	Town	1 km
UK04	M	>60	UK	Antibiotics	£8.20	City	5 km
UK05	M	>60	UK	for Angina	Free	Town	2 km
UK06	F	>60	UK	Sulfasalazine, Methotrexate	Free	Town	2 km
ZI01	F	20–40	Zimbabwe	Amoxycilin		Village	
ZM01	M	40–60	Zambia	Coartem	Free	Village	Close

**Table A2:** Interviewee summary.

Category	Value	Number of Interviewees
Sex	Male Female	11 19
Age range	<20 20–40 40–60 >60	1 12 10 7
World—developing	Total Of which: Egypt Kenya Kazakhstan Nigeria Tanzania Uganda Zambia Zimbabwe	24 1 14 1 1 4 1 1 1
World—developed	UK	6
Type of location	City Town Village	3 9 18
Medicine	Antibiotics Cough medicine Malaria medicine Painkillers Other	5 2 11 4 8
Medicine cost	Free <£1 £1–£2 £2–£3 >£3 Unstated	6 8 6 2 3 5
Distance to obtain	<1 km 1–2 km 3–4 km 5–6 km >7 km Unstated	8 11 3 4 2 2

## Appendix B. Interview Coding

Table A3 lists coding with sample interview content and references for the developing world. Table A4 lists the same for the developed world.

**Table A3:** Interview coding, developing world.

Transcription Code	Interview Example	Ref.
Distance, positive, close	…pharmacies in every street… just down the road from our flat But if I need to get it from a pharmacy it’s a km I walk, I take one minute to get to the health centre Not very far. Just walk to get them It was 2 km away 2 km from my home 2 km from my home 2 km from home About 1 km Pharmacy isn’t far, about 10-min walk from my house Just nearby. Two minutes Just a few meters… two minutes’ walk Not too far Only 10 min’ walk to the [small] pharmacy… when you want to go to the big pharmacy it takes about 15 min	EG01 KN03 KN05 KN06 KN08 KN09 KN11 KN15 KN14 KZ01 NG01 TZ02 TZ03 TZ04
Instructions, negative, foreign language, verbal	I don’t understand colloquial Arabic	EG01
Instructions, negative, foreign language, written	I think we figured out the written instructions …you really don’t understand the reading …people who can’t even read	EG01 EG01 EG01
Utensils, negative, missing	I don’t think there was a spoon. I think we had to buy it separately	EG01
People, positive, present	Probably my husband was there sometimes Mum and my younger sisters were there It’s better for someone to make sure you get the full dose Mother and brothers were there Grandmother was there with me as I have no parents I was with the physician only Family members With a friend My parents My wife is the one who was always reminding me to take it	EG01 KN04 KN06 KN09 KN11 KN13 KN14 KN15 KZ01 TZ03
Content, negative, unknown	…you really don’t have a clue what’s in it… [it’s] at the back of your head that it could be anything I don’t like taking medicine…because of the idea that it’s chemicals… natural ones are better than synthetic	EG01 NG01
Branding, positive, known	I suppose the branding just makes you trust it more	EG01
Motivation, negative, last resort	I think I sort of used it as a last resort Just like when I’m really sick, I’m like distressed for getting better… makes me take the pills Urge to get healed I was physically weak and mentally disturbed… I felt desperate Totally disturbed… Eager to know its [effect] Felt hard to use since I don’t like medicines I’d have taken anything	EG01 KN03 KN07 KN08 KN10 KN15 KZ01
Diagnosis, negative, foreign language, verbal	…would have helped if the person that we saw could speak English	EG01
Taste, negative, bad	Sometimes obviously the taste of the medicine …the taste of the drugs I don’t like it. I don’t like taking medicine because of the taste They don’t taste well when you swallow them. Bad taste [not completing the full dosage] is primarily caused by… difficulty in taking the medicine due to… taste… I took one but couldn’t take more because of the nasty taste	EG01 KN03 NG01 TZ03 ZI01 ZM01
Effects, negative, bad	…it’s not good for you… Sometimes it can harm the body …if I take the medicine it weakens my body for some time …in fact the body constitution was changed… The medicine itself was reactive… …the Coartem seems to be a bit too much for me I hear about these doctors saying about how conventional medicines affect the liver	EG01 KN10 KN15 TZ02 TZ02 ZM01 ZM01
Effects, negative, side, general	…there’s all these side effects… I don’t like taking medicine because… there’s side effects …taking tablets irritates them	EG01 NG01 TZ01
Beliefs, negative, others, too dependent	…“Paracetamol doesn’t work for you because you keep taking it” …so I’ll have to bargain for half a tablet of Paracetamol if my temperature is high as a kid, they didn’t believe in medicine much	EG01 KZ01
Beliefs, negative, profit, pharma	…this thing about the pharmaceutical industry and how they’re making profit	EG01
Beliefs, negative, profit, herbal	…the natural remedy people are also making their profit as well	EG01
Stop, negative, better	I wouldn’t even [complete the course] if the GP said “make sure you finish the course” …after 3 days you feel like you’re ok. You’re like, “No I don’t need to get more medicines then” Many people [stop when they feel better] Sometimes I’ll take it according to the prescription but sometimes I stop when I feel better Sometimes I feel that I’m feeling better When they see they’re a little better they stop taking the tabs …then I got well… feeling well before finishing the dose When one takes the medicine and gets better maybe he feels fine, so it’s difficult for him to finish the dose And some, when they feel better, then drop the medicine [not completing the full dosage] is primarily caused by early signs of healing… For some, I think the moment they feel better they choose not to take any more	EG01 KN03 KN06 NG01 TZ01 TZ01 TZ03 TZ04 TZ04 ZI01 ZM01
Cost, positive, low	At the hospital sometimes we don’t pay About 100 Tz Shillings [£0.03, $0.05] Ksh20 [£0.13, $0.20] Ksh70 [£0.47, $0.72] Ksh50 [£0.33, $0.52] Tsh1000 [£0.30, $0.46] We go to the hospitals. They give out malaria tablets for free For things like Coartem… they don’t really charge	KN03 KN01 KN05 KN12 KN14 TZ03 TZ04 ZM01
Reminder, positive, alarm	I use an alarm for night Some medicines I have to put alarm on reminding myself not to forget this	KN01 KZ01
Taste, negative, bitter	It’s… bitter I think there should be much… reduce the bitterness Some medicines are bitter this makes it hard to consume too… bitter Bitterness of the medicine… it is so bitter I hate medicine. They are bitter Reduce the bitterness… of the tabs It becomes easier to take if medicine is tasty… [Make them] a bit sweet Better something that is sweet Some are very, very… some are not sweet, you know. They’re so sour. I think if maybe sweeter, then somebody can swallow it easier And some, because the medicine is soooo bitter, drop it from taking the whole dose	KN01 KN01 KN07 KN09 KN11 KN12 KN04 KN07 KN09 NG01 TZ04 TZ04
Size, negative, big	It’s big… One is like the size of the pill This tablets are in large sizes and so swallowing becomes a problem The size is too big Size of this medicine is so big …at least the size of it should be moderate to make easier swallowing Reduce… the largeness of the tabs A bit… small[er] [not completing the full dosage] is primarily caused by… difficulty in taking the medicine due to… size… …you swallow them and it feels like you haven’t swallowed them and you wonder how you’re going to take the next tablets…	KN01 KN03 KN08 KN09 KN11 KN01 KN04 KN09 ZI01 ZM01
Formulation, negative, injection	I fear injections! I prefer medicines than the injection I prefer oral	KN01 KN03 KN07
Effects, negative, side, specific	I’ve read about side effects like your digestive system… Some people develop boils, others get sick, get weak, sweat a lot …now vomit… …I feel like vomiting …I could feel dizziness in me …they take medicines and end up vomiting …you become very tired It makes me feel so dizzy, a lot of noise in the ears, chilling of the body, loss of appetite, sometimes vomiting. This makes [me] feel bad, dodge the dosage …even produce a smell when urinating or on the skin or in sweat… Sick for a whole week and all that, the headaches, stomach stuff, the pains. I thought not to go through all that [by consuming the medicine]	EG01 KN01 KN03 KN06 TZ02 TZ02 TZ04 UG01 ZI01 ZM01
Taste, positive, sweet	The ones we have around here are very sugary so very easy for someone to take I liked it	KN03 KN14
Distance, negative, far	If I need to get from the hospital I have to go 4 km away 5 km from home. Travelled by Nissan at a cost of ksh400 [$3.95, £3] …good pharmacy shops are not available in the rural areas Almost 6 km 4 km from home 3 km from home. Used a motorbike which costed ksh200 [£1.33, $2.06] The problem is the pharmacy doesn’t open on Monday so we had to drive to her home about 3.5 km away 4 h [travel time] It’s 30 km to and from, to the pharmacy. $10 [£6.57] transport	KN03 KN04 KN07 KN10 KN12 KN13 KZ01 TZ01 UG01
Beliefs, negative, others, stigma	…when I’m there I’m not feeling comfortable to take the pills… so stigma itself can cause or make someone not to take the medicines… stigma is a major issue I sometimes I never just wanted to take medicine, because that I feared for stigma… sometimes when I wanted to take that medicine I could just hide People are afraid of that stigma… when people have HIV and AIDS they always try to hide it from people	KN03 TZ02 TZ02
Food, positive, present	Use of porridge Porridge I had eaten My mum was cooking Yes [I have food]. Normally you have to eat for medicine I do take it with… porridge	KN11 KN12 KN15 KZ01 NG01 TZ04
Food, negative, absent	If you don’t have something to eat you won’t take the drug… you have nothing to eat …take them after every meal. This was not possible due to poorness. We cannot afford 3 meals a day so it was hard to take the tabs in the afternoon… I did not take it at that time because I was hungry and tired No [I did not consume] I was hungry I wasn’t getting enough food… I really felt that drug if I hadn’t eaten It’s difficult to have enough food to visit the prescription We Africans take some medicines with not enough food They require a lot of drinks and eating well but we are poor we can’t afford most of the requirements. Sometimes we have a single meal a day	KN03 KN04 KN04 KN11 KZ01 TZ01 TZ01 UG01
Beliefs, negative, foreign origin	I don’t like taking medicine because… it’s foreign	NG01
Beliefs, negative, lack of faith	…if you don’t have that [faith to be healed] then you’ll have to take medicine	TZ02
Course, negative, long	…sometimes prescriptions take long time, many days for you to finish the dose I wished I could consume them once and over… I thought I would be given medicine to consume once and over… In general medicines are difficult for me to take. The dosage may be long It becomes easier to take medicine… does not taking too long To get relieved at once Others they are not following the information [from the doctor] They take long to heal, it’s a long dosage of 3–6 days	KN03 KN04 KN07 KN09 TZ04 UG01
Stop, negative, replaced by other	…maybe going for other drugs to see if they treat quicker… I end up not taking the other dose…	KN03
Stop, negative, keep	They act like emergency for my family I keep it just in case I get a re-occurrence of same symptom. Then I take the leftover when I cannot get to buy another Here in Africa, many people… keeping a dose…	KN03 NG01 TZ01
Motivation, positive, stay well	I don’t want to feel sick again tomorrow so I must complete the medicine If maybe I could default then I could have been maybe in danger In general I think it’s good for taking all malaria tabs because if you don’t… then you can feel worse when malaria attacks again	KN06 TZ02 TZ04
Motivation, positive, get well	Hopes came with the medicine… I used my illnesses as a reason to take it right away I knew soon I will be well	KN13 KN14
Effects, positive, others	Also, experience from other people. If maybe my [family] used the same drug and she got well, definitely that helps me to finish…	KN03
Regimen, negative, unacceptable	You realise it’s hard for me to wake up in the midnight to take pills Personally I go for prescription guidelines [as cause of failure]. They easily make me not to finish the prescription And with the tablets, they feel like there’s too many	KN03 KN03 ZM01
Cost, negative, high	Ksh500 [$4.95, $3.75] was the cost of the medicine Ksh150 [£1, $1.53] Ksh130 [£0.87, $1.33] Ksh200 [£1.34, $2.05] Ksh450 [£3.01, $4.60] Ksh300 [£2, $3.09] to buy the medicine Ksh250 [£1.67, $2.57] Fairly expensive for Kazakhstan…about £3–4… they tend to look at how you’re dressed 450 Nira [£1.49, $2.27] …malaria medicine is not affordable to a lot of people… Tsh2000, 5000 [£0.58, $0.91; £1.46, $2.27] depending on the quantity … but mainly in hospitals there are less malaria tabs so most people go to buy them in the pharmacy… there are some tablets from India, there are some tabs from Western countries and then there are some tablets from the local, from within the country. So within the country you can find them at tsh1000 [£0.29, $0.45]. And then tabs from outside the country goes to tsh3000 [£0.88, $1.36] to tsh5000 [£1.47, $2.27] …some cannot afford the full dose $6 [£3.94] medicine	KN04 KN08 KN09 KN10 KN11 KN13 KN15 KZ01 NG01 NG01 TZ02 TZ04 TZ04 UG01
Instructions, negative, misunderstood	I know how to take Coartem… we take two tabs, two times a day	KN05
Instructions, positive, clear, verbal	They explained it clearly how to take it I knew… by listening My teacher told me to follow the doctor’s prescription …the doctor showed me the correct way I just listened to a doctor so that I can follow what he has told me I followed the instruction given to me by the doctor I realised its importance… after being taught the effects of that medics when taken wrongly	KN05 KN07 KN11 KN14 TZ01 TZ03 UG01
Course, positive, acceptable	I take it up to the last one I take it until I use all the tablets I do follow the information	KN05 NG01 TZ04
Effects, negative, others	I just see them, they want to go vomit	KN06
Stop, negative, discarded	They throw it away, because you can’t go on taking the medicine	KN06
Access, negative, hard	…with curative I found after going to various pharmacy shops I did not obtain the medicine [until]… the third shop	KN07 KN08
Formulation, positive, liquid	Personally I would go for liquid People around here with children they like syrups If they can convert this tabs into syrup… the better	KN03 KN07 KN08
Regimen, negative, unexpected	I could not actually imagine there will be a prescription or directive on how to take the medicine… I thought I could just… consume regardless… I thought I will get better at that moment I get a medicine to drink once and get cured I had planned to take large amounts It was not in my plan to consumer it according to the prescriptions…	KN08 KN11 KN13 KN14 UG01
Water, negative, absent	The medicine was to be consumed… with a lot of water which I did not have sufficient of… I lacked water… I was thinking of taking the medicine without water	KN08
People, negative, absent	There was no body… No [I did not consume] On my own… No, I stopped [not completing the full dosage] is primarily caused by… difficulty in taking the medicine due to… lack of monitoring of the sick by fit family members	KN08 TZ04 ZI01
Smell, negative, bad	This medicine has a smell and this smell surely disturbs me a lot when taking the medicine Some medicines do emit a pungent smell that will cause nausea and vomiting… [Is the smell sufficient to stop taking?] Yes bro absolutely! As soon as you open the package you actually feel the strong smell	KN08 ZI01
Beliefs, positive, confidence	I had confidence that it will relieve my pain	KN09
Water, positive, present	Water helped me to consume Water… helped Water …with a lot of water. Yes, I have enough water I do take it with tea… Yes, yes. I have access Yes, my eldest sister, they take their medicine with Coca-Cola	KN09 KN11 KN12 TZ03 TZ04 ZM01 ZM01
Formulation, positive, injection	[Easier] through syringe I prefer the injection before because I don’t like the taste of medicine …in the east region [of Africa] there are some people… the majority… who prefer injections… The other [sister], they prefer the injections to tablets	KN09 NG01 TZ02 ZM01
Beliefs, positive, others	I had been informed about its advantages	KN10
Instructions, negative, unclear, written	So even though the packaging said something else, the doctor specified “something something 3 times”. I had to ask my parents to decode the curvier writing. [without that] it would have been a bit of a guess	KZ01
Regimen, positive, acceptable	I didn’t mind for instance at night-time to wake up	KZ01
Regimen, negative, complex	[Prefer] once per day [Prefer to] take many dosage for a quick recovery I would like to take it whenever I go to bed I had to make sure that they eat in the morning… the first two tablets of the day were regular and then not When I go to the clinic, I just get the diagnosis and I go for other medications… there were too many tablets. So I took my pawpaws and I was ok in 2 days. The malaria was all gone	KN12 KN14 KN15 KZ01 ZM01
Regimen, negative, forgot	And then once I forgot, I misplaced it, so I missed it The time I forgot to take it. I repeated the dose that I did not take	KZ01 TZ03
Instructions, negative, unclear, verbal	So it was a very vague direction so I didn’t assume that it was critical	KZ01
Routine, negative, absent	…if your day gets mixed up with night and you’re really not sure any more what to stick to That occurs so much in Africa! Maybe you can miss in that case in the evening, or forget in the morning and then take in the afternoon then miss in the evening, or someone can take 6 at once! …some people I know only take them in the night	KZ01 TZ04 TZ04
Routine, positive, present	I tend to be pedantic about those things… I’ve been given a task… I’m going to do this… I might as well do it properly I try as much as possible to get it at home. After my meal, my breakfast, and when I return from work I make sure that I am in the house I just started following the prescription strictly… I was at home I remember if I want to eat I have to take medicine [Are you always at home?] Yes, it is	KZ01 NG01 TZ01 TZ02 TZ03 TZ04
Cost, negative, herbal, low	…the herbal [malaria medicines] are very cheap …medicines from China… food supplement… cheaper Or if you don’t have money you just can take some local medicine	NG01 NG01 TZ04
Beliefs, negative, value	Sometimes they say that the tablets are weak	TZ01
Stop, negative, busy	I was occupied maybe from work Because maybe they’re occupied	TZ03 TZ03
Storage, negative, unsafe	…maybe the people being lazy can just put them where children are reaching and then the children can consume them… it can be more dangerous	TZ04
Stop, negative, run out	…some cannot afford the full dose	TZ04

**Table A4:** Interview coding, developed world.

**Transcription Code**	**Interview Example**	**Ref.**
Distance, positive, close	Walk… We don’t live too far away, about half a mile 10 yards. The doctor’s and the chemist’s are together About a quarter of a mile About a mile	UK02 UK03 UK04 UK05 UK06
People, positive, present	[What made applying it possible?] Someone else did it Obviously have breakfast together and dinner… …with the family I took the responsibility on so she didn’t have to think about it Yes. “Have you taken your tablets?”	UK01 UK02 UK04 UK04 UK05
Content, negative, unknown	I wouldn’t want to be putting a lot of stuff into my body that I didn’t know what it was doing	UK06
Motivation, negative, last resort	I never want to take drugs… only because he said to take them I took them I was sad that I was prescribed it for the illness I was said to have, but I took it	UK04 UK05
Stop, negative, better	I don’t take the prescribed dose every day… I can go a fortnight without taking them… when I haven’t got the symptoms I’ll knock them… I’ll take them for several days until I notice it’s subsided and then I’ll stop	UK05 UK05
Cost, positive, low	[They’re all free?] Yes [It didn’t cost you anything?] No Fortunately [wife] had an exemption… Free [You don’t have to pay?] No	UK01 UK03 UK04 UK05 UK06
Instructions, positive, clear, written	[Easy to understand?] Yes It was written on the doctor’s prescription. And a copy on the packet I think the label on the tablet bottle said that …it has a little leaflet inside Because it was on the box that the tablets came in	UK01 UK03 UK04 UK05 UK06
Size, negative, big	The Sulfasalazine are quite large and hard but no, no problem… just the size, but as long as my tea is not too hot	UK06
Food, negative, absent	Sometimes when I remembered there wasn’t another chance to eat	UK01
Stop, negative, keep	I don’t feel any ill effects by not taking them… I’ve got those in stock that I can draw on if I need	UK05
Motivation, positive, stay well	I don’t want to have any problem coming up because I’ve forgotten to take them or decided not to take the medicine he’s prescribed. That would be foolish And from starting to take those tablets I have had no swelling and no pain. I still take them I was extremely grateful that there was something I was being given to keep down the… pain, and it did I don’t want to risk a return to the swelling and pain… I would not risk stopping taking them	UK02 UK06 UK06 UK06
Motivation, positive, get well	[Positive results encouraged you to carry on?] Yes I was happy because it would take away a lot of the pain The results were absolutely magical, marvellous, a miracle	UK03 UK04 UK06
Regimen, negative, unacceptable	I didn’t put it on my back very often because it was hard to get to… I had to clean it before, so that was annoying as well	UK01
Cost, negative, high	Yes, £7 or whatever	UK04
Instructions, negative, misunderstood	It said take 2 twice a day but I didn’t know what that meant	UK01
Instructions, positive, clear, verbal	I think he must have said “take one per day”, which I did every morning I was told how to take them	UK03 UK05
Course, positive, acceptable	[Take in accordance with the prescription?] Yes	UK01
Water, negative, absent	Sometimes. Not always	UK01
People, negative, absent	[And when you didn’t apply it you were on your own?] Yes	UK01
Water, positive, present	[…take them all with water?] Yes I took it with a drink …with a cup of tea Water …with a cup of tea	UK02 UK03 UK04 UK05 UK06
Regimen, positive, acceptable	…breakfast time is set and teatime is set so twice a day fits in quite happily with that I didn’t need to take one 3 times a day. I could take the 3 at breakfast time	UK04 UK06
Regimen, negative, complex	I had to take it with food 8 h apart, an hour before I ate…I had to take it during the gap between my lessons before lunch but that’s actually 50 min… and then on the bus as soon as I got on, for tea… there were a lot of times I actually forgot [If you had a choice of how to take…?] I’d say not with food Especially the hour before food, you don’t know when you’re next going to have food …it was a real concoction of working out what she needed at each time so I devised a spreadsheet It was something that sounds simple but was such an onerous task day after day You might have run out of 50 s but you’ve got 25 s so you give three 25 s or combinations of… it was an absolute logistical nightmare	UK01 UK01 UK01 UK04 UK04 UK04
Regimen, negative, forgot	Perhaps very very occasionally if we’ve been out to a late dinner… I might have forgotten Well very rarely	UK02 UK03
Instructions, negative, unclear, verbal	…and the pharmacist might have grunted that at me as he passed it over Initially, yes, but everything was so fluid… that it became evident that it didn’t really matter too much	UK04 UK04
Routine, negative, absent	…change in routine, like on a weekend… or I was staying in someone’s house, I’d forget to take it …but if we ate upstairs or in a different room I wouldn’t take it	UK01 UK01
Routine, positive, present	…one in the morning and one at night. Getting up and going to bed. Part of the routine… Just sort of when getting up or going to bed it jogged my memory I put it in the dining room because I had to take it with a meal I take certain ones with a drink with my breakfast or before my breakfast, and I have some… in the evening also before I take a drink I fill the containers… for 7 days… [then] I don’t forget them… I’m capable of remembering what should be in each I always took the packet out and took it with my breakfast So it was quite easy as long as I’d got them with me In the morning with breakfast with a cup of tea… evening meal again with a cup of tea In a morning [At breakfast?] Yes [Do you have them in a box with flaps?] Yes. [Does that help?] Very much so I got a little box with a week of separated compartments… I don’t have to think about it in a morning At the breakfast table	UK01 UK01 UK01 UK02 UK02 UK03 UK03 UK04 UK05 UK05 UK06 UK06
Stop, negative, run out	We had to eke them out instead of having like 2 tablets twice a day we had to have 1…	UK04
Access, positive, easy	Mum picked it up Walk, or perhaps drive in if I’m going to town… it’s a standing order… it’s very simple Collected from Boots… they have an arrangement by which you collect regular medicines [It wasn’t inconvenient?] No We just go and pick it up from the chemist It could be delivered to me but I’m usually out… so I call	UK01 UK02 UK03 UK03 UK05 UK06
Motivation, negative, tired	[When you didn’t apply it, you were…?] Tired	UK01
Beliefs, negative, pointless	There didn’t seem to be a lot of point [in consuming]… I don’t know really what I’m taking tablets for… I doubt his diagnosis actually… If I’ve no pain then I don’t need it preventing	UK01 UK05
Reminder, positive, general	Some kind of reminder, especially when I’m staying over	UK01
Instructions, positive, compliant	I have been advised by my doctor to take these… and therefore I’m quite happy to take whatever he has prescribed… I just do as I’m asked to do	UK02 UK06
Formulation, positive, tablet	No it was very simple as it is, in foil In my case, no. They’re just tablets [wife] was always very good at swallowing tablets I find tablets pretty easy [What you’ve got is fine?] Yes	UK03 UK04 UK04 UK05 UK06
Size, positive, small	[Any problems?] No. [Small enough?] Swallow them down	UK05
Effects, positive, side, none	[Any side effects?] Not to my knowledge	UK06
